# Real-time loop-mediated isothermal amplification: an early-warning tool for quarantine plant pathogen detection

**DOI:** 10.1186/s13568-019-0774-9

**Published:** 2019-04-24

**Authors:** Chiara Aglietti, Nicola Luchi, Alessia Lucia Pepori, Paola Bartolini, Francesco Pecori, Aida Raio, Paolo Capretti, Alberto Santini

**Affiliations:** 1 Institute for Sustainable Plant Protection, National Research Council (IPSP-CNR), Via Madonna del Piano 10, 50019 Sesto Fiorentino, Firenze Italy; 2 Department of Agrifood Production and Environmental Sciences (DISPAA), University of Florence, Piazzale delle Cascine 28, 50144 Firenze, Italy

**Keywords:** Alien pathogens, Canker Stain Disease, Isothermal amplification, LAMP, Olive Quick Decline Syndrome, Portable diagnostics, Sudden Oak Death

## Abstract

An effective framework for early warning and rapid response is a crucial element to prevent or mitigate the impact of biological invasions of plant pathogens, especially at ports of entry. Molecular detection of pathogens by using PCR-based methods usually requires a well-equipped laboratory. Rapid detection tools that can be applied as point-of-care diagnostics are highly desirable, especially to intercept quarantine plant pathogens such as *Xylella fastidiosa*, *Ceratocystis platani* and *Phytophthora ramorum*, three of the most devastating pathogens of trees and ornamental plants in Europe and North America. To this aim, in this study we developed three different loop mediated isothermal amplification (LAMP) assays able to detect each target pathogen both in DNA extracted from axenic culture and in infected plant tissues. By using the portable instrument Genie^®^ II, the LAMP assay was able to recognize *X. fastidiosa*, *C. platani* and *P. ramorum* DNA within 30 min of isothermal amplification reaction, with high levels of specificity and sensitivity (up to 0.02 pg µL^−1^ of DNA). These new LAMP-based tools, allowing an on-site rapid detection of pathogens, are especially suited for being used at ports of entry, but they can be also profitably used to monitor and prevent the possible spread of invasive pathogens in natural ecosystems.

## Introduction

Invasive alien species represent a primary threat to biodiversity, economy and human health. International trade, tourism and other human activities break geographical barriers introducing non-native pathogenic organisms into new environments where they eventually find susceptible hosts and environments (Fisher et al. 2012; Migliorini et al. 2015; Santini et al. 2018). In Europe the accidental introduction of three quarantine pathogens, *Xylella fastidiosa*, *Ceratocystis platani* and *Phytophthora ramorum* with infected plants or wood material, has led to epidemics with heavy economic and ecological impacts.

*Xylella fastidiosa* is a bacterium reported on more than 350 different hosts (Denancè et al. 2017) and since 2013 is responsible for Olive Quick Decline Syndrome in Southern Italy (Apulia) (Saponari et al. 2013), more recently it has been found in Tuscany (Central Italy) (EPPO 2019); *Ceratocystis platani* is an ascomycetous fungus reported as the causal agent of Canker Stain Disease (CSD) of plane tree (*Platanus*) in urban and natural ecosystems (Lehtijärvi et al. 2018; Tsopelas et al. 2017). *Phytophthora ramorum* is an oomycete causing Sudden Oak Death (SOD) in the USA (Rizzo et al. 2002) but the pathogen has also been found in European ornamental nurseries (Werres et al. 2001) and in plantations of Japanese larch (*Larix kaempferi*) in Great Britain (Brasier and Webber 2010).

In the last decades alien plant pathogens are exponentially establishing in Europe (Santini et al. 2013). The European Union (EU) has an open-door phytosanitary system, which means that plants not specifically regulated can enter, therefore, inspections are concentrated on well-known pests and mostly limited to visual examination of aerial parts of plants. Traditional inspection methods are time consuming and labor-intensive, requiring specialized laboratories and expert operators. Furthermore, the first disease symptoms can occur after a long latent phase of the infection and they may be non-specific (e.g. *X. fastidiosa*), hampering detection efforts and, therefore, timely management of potential outbreaks. Serological and immunoassay-based methods are available, but their low sensitivity and specificity make them unreliable for phytosanitary inspections. For these reasons, sensitive and specific tools for effective phytosanitary inspection and interception are required to prevent new pathogen introductions. Nowadays, the high specificity and sensitivity of molecular DNA-based technologies allows detection of pathogens in the early stages of infection, when they are present at low DNA concentrations (Bilodeau et al. 2007; Chandelier et al. 2006; Harper et al. 2010; Luchi et al. 2013; Rollins et al. 2016). Although many of these methods have been used routinely in the laboratory, most of them are not transferable for field inspection, seriously limiting their adequacy for point-of-care application (Lau and Botella 2017). Point-of-care methods, besides being sensitive and specific, should also be simple and fast, producing results that are easy to interpret and demanding minimal equipment and facilities (Tomlinson et al. 2010a). For these purposes, an affordable LAMP (Loop mediated isothermal amplification) technique (Notomi et al. 2000), seems to be the most suitable. Recently several LAMP assays have been developed for both field and lab use especially for human and animal diseases and food safety control (Abdulmawjood et al. 2014; Lucchi et al. 2010). Up to now, even if many LAMP-based assays were developed for plant pathogens (Chen et al. 2013; Dai et al. 2012; Hansen et al. 2016; Harper et al. 2010; Moradi et al. 2014; Peng et al. 2013; Sillo et al. 2018; Tomlinson et al. 2007), only a few tests (Bühlmann et al. 2013; Franco Ortega et al. 2018; Harrison et al. 2017; Tomlinson et al. 2010b, 2013) were optimized and applied on portable instrument for on-site use. The use of portable detection instruments is a major driving force to achieve point-of-use, and real-time monitoring of analysed samples, allowing rapid detection.

The aim of this study was to optimize a reliable, fast and sensitive diagnostic assay using a LAMP portable instrument for early detection of *X. fastidiosa*, *C. platani*, and *P. ramorum.* These new protocols will be available to be used for research aims and for phytosanitary inspection, in order to prevent further introductions and spread of these pathogens.

## Materials and methods

### Microbial strains and DNA extraction

In addition to the targeted pathogens, fungal and bacterial species phylogenetically related to target pathogens, as well as out-group species and common host colonizers were used to optimize the molecular assay (Table 1).

**Table 1:** List of isolates used in this study

Species	Isolate code	Group^b^	Host	Origin	Collector^c^	Molecular assay^d^		
						LAMP^e^		qPCR^f^
						t_amp_ (min:s)	Ta (°C)	Detection
*X. fastidiosa assay*								
*Xylella fastidiosa* subsp. *pauca*	Co.Di.Ro^a^	T	*Olea europaea*	Italy	M. Saponari	7:15	88.98	+
*Xylella fastidiosa* subsp. *fastidiosa*	Xff^a^	T	*Prunus dulcis*	USA	J. Chen	14:20	88.78	+
*Xylella fastidiosa* subsp. *multiplex*	Xfm^a^	T	*Liquidambar styraciflua*	USA	S. Russell	7:00	88.83	+
*Pseudomonas savastanoi* pv. *savastanoi*	ITM05^a^	CHC	*Olea europea*	Italy	G. Marchi	–	–	−
*Pantoea agglomerans*	PaFL1^a^	CHC	*Olea europea*	Italy	G. Marchi	–	–	−
*Pseudomonas fluorescens*	KL218^a^	CHC	*Actinidia deliciosa*	Italy	G. Marchi	–	–	−
*Xanthomonas arboricola* pv. *pruni*	Xap	PR	*Prunus laurocerasus*	Italy	A. Raio	–	–	−
*Pseudomonas savastanoi* pv. *nerii*	Ps.sav	CHC	*Nerium oleander*	Italy	A. Raio	–	–	−
*Pseudomonas koreensis*	KL217^a^	NP, O	*Actinidia deliciosa*	Italy	G. Marchi	–	–	−
*Pseudomonas syringae*	KL34^a^	O	*Actinidia deliciosa*	Italy	G. Marchi	–	–	−
*Pseudomonas syringae*	KL32^a^	O	*Actinidia deliciosa*	Italy	G. Marchi	–	–	−
*Pseudomonas viridiflava*	KL24^a^	O	*Actinidia deliciosa*	Italy	G. Marchi	–	–	−
*Pseudomonas mediterranea*	C5P1rad1^a^	O	*Chrysanthemum* sp.	Italy	M. Fiori	–	–	−
*Pseudomonas corrugata*	C2P1 rad^a^	O	*Chrysanthemum* sp.	Italy	M. Fiori	–	–	−
*Pseudomonas syringae* pv. *photiniae*	CFBP2899^a^	O	*Photinia glabra*	Japan	CFBP	–	–	−
*Pectobacterium carotovorum*	C24^a^	O	*Zantedeschia aethiopica*	Italy	G. Marchi	–	–	−
*Pectobacterium carotovorum*	C6^a^	O	*Zantedeschia aethiopica*	Italy	G. Marchi	–	–	−
*Pantoea agglomerans* pv. *gypsophilae*	Ehg824-1^a^	O	*Gypsophila paniculata*	Israel	G. Marchi	–	–	−
*Pseudomonas syringae* pv. *actinidiae*	KL103^a^	O	*Actinidia deliciosa*	Italy	G. Marchi	–	–	−
*Pseudomonas syringae* pv. *tabaci*	GSPB1209^a^	O	*Nicotiana* sp.	Italy	G. Marchi	–	–	−
*Pseudomonas syringae* pv. *viburni*	CFBP1702^a^	O	*Viburnum* sp.	USA	CFBP	–	–	−
*Sphingomonas* sp.	KVPT7FA^a^	NP, CHC	*Actinidia deliciosa*	Italy	G. Marchi	–	–	−
*Stenotrophomonas malthopylia*	St	NP, PR	*Capsicum annum*	Italy	A. Raio	–	–	−
*Xanthomonas campestris* pv. *campestris*	Xcc	PR	*Brassica* spp.	Italy	A. Raio	–	–	−
*Xanthomonas arboricola* pv*. juglandis*	Xaj	PR	*Juglans regia*	Italy	A. Raio	–	–	−
*Erwinia amylovora*	Ea12	O	*Pyrus communis*	Italy	C. Cainelli	–	–	−
*Agrobacterium tumefaciens*	LMG37	O	*Prunus* spp.	USA	BCCM/LMG	–	–	−
*Agrobacterium vitis*	CFBP5523	O	*Vitis vinifera*	Australia	CFBP	–	–	−
*C. platani* assay								
*Ceratocystis platani*	CBS117355	T	*Platanus* sp.	France	CBS-KNAW	10:08	88.14	+
*Ceratocystis platani*	Cp3	T	*Platanus* × *acerifolia*	Italy	IPSP-CNR	12:15	88.55	+
*Ceratocystis platani*	Cp6	T	*Platanus* × *acerifolia*	Italy	IPSP-CNR	9:08	88.30	+
*Ceratocystis platani*	G160	T	*Platanus* × *acerifolia*	Turkey	T. Dogmus	16:58	88.93	+
*Ceratocystis platani*	DB203	T	*Platanus* × *acerifolia*	Turkey	T. Dogmus	14:08	88.78	+
*Ceratocystis platani*	CBS115162	T	*Platanus occidentalis*	USA	CBS-KNAW	8:00	88.83	+
*Ceratocystis platani*	Cp24	T	*Platanus* × *acerifolia*	Italy	IPSP-CNR	8:00	88.43	+
*Ceratocystis fimbriata*	CBS 115167	PR	*Syngonium* sp.	USA	CBS-KNAW	10:05	88.45	−
*Ceratocystis fimbriata*	CBS 118126	PR	*Syzgium aromaticum*	Sulawesi	CBS-KNAW	15:10	88.70	−
*Ceratocystis fimbriata*	CBS 115175	PR	*Mangifera indica*	Brazil	CBS-KNAW	9:30	88.20	−
*Ceratocystis fimbriata*	CBS 115174	PR	*Eucalyptus* sp.	Brazil	CBS-KNAW	14:00	88.50	−
*Ceratocystis fimbriata*	CBS 115171	PR	*Colocasia esculenta*	Brazil	CBS-KNAW	10:13	88.60	−
*Ceratocystis fimbriata*	CBS 74040	PR	*Crotolaria juncea*	Brazil	CBS-KNAW	13:38	88.00	−
*Sarcodontia pachyodon*	Sp5	O, CHC	*Platanus* × *acerifolia*	Italy	IPSP-CNR	–	–	−
*P. ramorum* assay								
*Phytophthora ramorum*	Pram^a^	T	*Rhododendron* sp.	Greece	P. Tsopelas	7:58	88.68	+
*P. ramorum*	LSVM123^a^	T	*Rhododendron* sp.	France	R. Ioos-N. Schenck	7:07	88.48	+
*P. ramorum*	LSVM362^a^	T	*Rhododendron* sp.	France	R. Ioos-N. Schenck	6:15	88.73	+
*P. ramorum*	LSVM386^a^	T	*Rhododendron* sp.	France	R. Ioos-N. Schenck	6:72	88.68	+
*P. ramorum*	LSVM390^a^	T	*Rhododendron* sp.	France	R. Ioos-N. Schenck	7:00	88.53	+
*P. ramorum*	LSVM391^a^	T	*Leucothoe* sp.	France	R. Ioos-N. Schenck	6:30	88.53	+
*P. ramorum*	LSVM401^a^	T	*Rhododendron* sp.	France	R. Ioos-N. Schenck	7:07	88.53	+
*P. ramorum*	LSVM402^a^	T	*Rhododendron* sp.	France	R. Ioos-N. Schenck	7:00	88.53	+
*P. ramorum*	LSVM405^a^	T	*Rhododendron sp.*	France	R. Ioos-N. Schenck	7:15	88.48	+
*P. lateralis*	Plat^a^	PR	*Chamaecyparis lawsoniana*	France	C. Robin	9:00	88.43	+
*P. alni* subsp*. uniformis*	Ph68	PR	*Alnus cordata*	Italy	G. P. Barzanti	23:27	90.47	−
*P. cactorum*	PCA1^a^	PR	*Aesculus hippocastanum*	Germany	J. Schumacher	–	–	−
*P.* × *cambivora*	Ph21^a^	PR	*Castanea sativa*	Italy	A. Vettraino	25:22	90.67	−
*P. cinnamomi*	28SA	PR	*Laurus nobilis*	Italy	IPSP-CNR	16:37	89.62	−
*P. cinnamomi*	Ncfc^a^	PR	Unknown	Italy	IPSP-CNR	–	–	−
*P. cryptogea*	13SA	PR	*Prunus laurocerasus*	Italy	IPSP-CNR	22:07	89.78	−
*P. citricola*	51RC	PR	*Viburnum lucidum*	Italy	IPSP-CNR	–	–	−
*P. citricola*	Pcl1^a^	PR	Unknown	Germany	T. Jung	27:27	89.82	−
*P. citrophthora*	33SB	PR	*Euonymus* spp.	Italy	IPSP-CNR	20:05	89.23	−
*P. citrophthora*	Ph9^a^	PR	*Convolvolus* sp.	Italy	S. O. Cacciola	18:15	89.58	−
*P. europaea*	PE1^a^	PR	Unknown	Germany	T. Jung	15:12	90.42	−
*P. foliorum*	2015–1454^a^	PR, CHC	*Rhododendron*	UK	A. Pérez-Sierra	11:15	89.08	−
*P. gonapodyides*	PG7^a^	PR	*Quercus robur*	Germany	S. Leonhard	–	–	−
*P. gonapodyides*	IHTM	PR	*Alnus cordata*	Italy	IPSP-CNR	–	–	−
*P. megasperma*	Ph78	PR	*Prunus avium*	Italy	G. P. Barzanti	–	–	−
*P. megasperma*	PMI^a^	PR	*Quercus robur*	Germany	S. Leonhard	–	–	−
*P. nicotianae*	1RB	PR	*Myrtus communis*	Italy	IPSP-CNR	–	–	−
*P. palmivora*	44RC	PR	*Prunus laurocerasus*	Italy	IPSP-CNR	–	–	−
*P. quercina*	PQ4^a^	PR	*Quercus robur*	Germany	S. Leonhard/J. Schumacher	–	–	−
*P. syringae*	Psy2^a^	PR	Unknown	Germany	J. Schumacher	17:45	89.48	−
*Elongisporangium anandrum*	PYA^a^	O	*Quercus robur*	Germany	S. Leonhard	–	–	−
*Phytopythium litorale*	40SB	O	*Prunus laurocerasus*	Italy	IPSP-CNR	–	–	−
*Elongisporangium undulatum*	76SB	O	*Cupressus sempervirens*	Italy	IPSP-CNR	–	–	−
*Mortariella* sp.	26RA	O	*Arbutus unedo*	Italy	IPSP-CNR	–	–	−
*Diplodia mutila*	Dm	O	*Quercus* spp.	Italy	IPSP-CNR	–	–	−
*D. pinea*	128	O	*Pinus resinosa*	USA	M. A. Palmer	–	–	−
*D. scrobiculata*	124	O	*Pinus resinosa*	USA	M. A. Palmer	–	–	−
*D. seriata*	UCD 352	O	*Vitis vinifera*	USA	J. R. Urbez-Torres	–	–	−
*D. seriata*	WP-J10	O	*Vitis vinifera*	Australia	S. Savocchia	–	–	−
*Geosmithia pallida*	IVV7	O	*Ulmus* spp.	Italy	IPSP-CNR	–	–	−
*Ophiostoma novo ulmi* subsp. *americana*	HI72	O	*Ulmus* spp.	USA	IPSP-CNR	–	–	−

^a^Samples provided as DNA

^b^For each molecular assay developed in this study different groups of isolates were tested: target species (T); phylogenetically related species (PR), CHC = common host colonizers species (CHC); out-group species (O)

^c^*CBS* Centraalbureau voor Schimmelcultures (CBS) Fungal Biodiversity Centre—Royal Netherlands Academy of Arts and Sciences (KNAW), Utrecht, The Netherlands; *CFBP* French Collection of Plant Pathogenic Bacteria, INRA, France; *IPSP-CNR* Institute for Sustainable Plant Protection—National Research Council, Firenze, Italy; *BCCM/LMG* Bacteria Collection Laboratorium voor Microbiologie Universiteit Gent Bacteria Collection, Laboratorium voor Microbiologie, Universiteit Gent, Belgium

^d^Molecular assays are referred to LAMP and qPCR described in Table 2

^e^*t*_*amp*_ amplification time, *T*_*a*_ annealing temperature, – not detected

^f^+ positive, − negative

Mycelium of fungal and oomycete isolates (stored at 4 °C in the IPSP-CNR collection) was grown on 300PT cellophane discs (Celsa, Varese, Italy) on potato dextrose agar (PDA; Difco, Detroit, MI, USA) in 90 mm Petri dishes and maintained in the dark at 20–25 °C according to species requirements. After 7–10 days the mycelium was scraped from the cellophane surface and stored in 1.5 mL microfuge tubes at − 20 °C.

Bacterial strains (stored at − 80 °C in the IPSP-CNR collection) were grown on Luria–Bertani (LB) agar for 24 h at 25 ± 2 °C. Single colonies were picked-up and transferred to tubes containing 5 mL of LB that were incubated in an orbital shaker at 25 ± 2 °C and 90 rpm overnight. One millilitre of each suspension was used for DNA extraction. Fungal and oomycete DNA suitable for molecular analysis was extracted from mycelium by using the EZNA Plant DNA Kit (Omega Bio-tek, Norcross, GA, USA), as described by Migliorini et al. (2015). DNA from bacteria was extracted by using EZNA Bacteria DNA Kit (Omega Bio-tek) according to the procedure described by the manufacturer. DNA from the quarantine pathogens *X. fastidiosa*, *E. amylovora*, *P. ramorum* and *P. lateralis* were kindly provided by different collectors (see Table 1). Concentration of extracted DNA was measured using a Nanodrop ND-1000 spectrophotometer (NanoDrop Technologies, Wilmington, DE, USA).

### Plant DNA samples

Plant samples were analyzed from naturally infected hosts including: (i) Two symptomatic plants of each of the following Mediterranean maquis species were collected in March 2019: *Rhamnus alaternus*, *Calicotome spinosa*, *Cistus incanus*, *Spartium junceum*, *Prunus dulcis*, affected by *X. fastidiosa* subsp. *multiplex* (recently detected by Tuscany Regional Phytosanitary Service—EPPO 2019); (ii) 10 *Platanus* × *acerifolia* symptomatic trees infected by *C. platani* (Florence, Italy).

About 80 mg (fresh weight) of plant material, i.e. leaves of Mediterranean maquis species and wood of *P.* × *acerifolia* plants, were used for genomic DNA extraction by using two different extraction protocols: (i) on-site by using Plant Material DNA extraction kit (OptiGene), according to manufacturer’s instructions. Briefly, small pieces of plant material (c.a. 80 mg) were placed in a 5 mL bijou with ball bearing and 1 mL lysis buffer. Bijous were shaken vigorously for 1 min to ground the plant material. Plant material solution (10 μL) was transferred into a vial containing 2 mL dilution buffer and mixed. Finally, 3 μL of dilution buffer containing DNA has been used as template in a LAMP assay;

ii) in laboratory by using EZNA Plant DNA Kit (Omega Bio-tek). Plant material of all the collected samples for DNA extraction was transferred to 2 mL microfuge tubes with two tungsten beads (3 mm) (Qiagen) and 0.4 mL lysis buffer P1 EZNA Plant DNA Kit (Omega Bio-tek, Norcross, GA, USA) then ground with a TissueLyser (Qiagen) (30 oscillations/s for 1 min). DNA was extracted from all samples using the EZNA Plant DNA Kit (Omega Bio-tek) (Migliorini et al. 2015).

In addition to the above samples, the optimization of LAMP assay was conducted by using the following DNA samples stored at − 80 °C (IPSP-CNR DNA collection): (i) 10 DNA samples extracted from symptomatic *Olea europaea* leaves with *X. fastidiosa* subsp. *pauca* infections. DNA was kindly provided by M. Saponari (IPSP-CNR, Bari) and extracted in CTAB buffer (Loconsole et al. 2014); (ii) 10 DNA samples from symptomatic *Viburnum tinus* leaves affected by *P. ramorum* extracted by using EZNA Plant DNA Kit (Omega Bio-tek).

As negative control, fresh tissue collected from 10 healthy plant of each tested species (*Olea europaea*, *Rhamnus alaternus*, *Calicotome spinosa*, *Cistus incanus*, *S. junceum*, *Prunus dulcis*, *Platanus* × *acerifolia* and *Viburnum tinus*) were extracted by using both Plant Material DNA extraction kit (OptiGene) and EZNA Plant DNA Kit (Omega Bio-tek), as previously described.

### LAMP primer design

The six LAMP primers included: two outer primers (forward primer, F3; backward primer, B3) two inner primers (forward inner primer, FIP; backward inner primer, BIP) and two loop primers (forward loop primer, FLP; backward loop primer, BLP), as required by LAMP reaction (Notomi et al. 2000). Primers were designed using LAMP Designer software (OptiGene Limited, Horsham, UK) (Table 2) on the basis of the consensus sequences of the ribosomal RNA gene (ITS1-5.8 S-ITS2) for *P. ramorum* (KC473522) and *C. platani* (EU426554.1), while for *X. fastidiosa* the ribosome maturation factor (RimM) gene belonging to Co.Di.Ro strain was chosen (JUJW01000001). All designed primers were synthesized by MWG Biotech (Ebersberg, Germany) and are reported in Table 2. The specificity of newly designed primers was further tested using nucleotide–nucleotide BLAST^®^ (Basic Local Alignment Search Tool; http://www.ncbi.nlm.nih.gov/BLAST) (Altschul et al. 1990).

**Table 2:** List of primer set used in this study

Target species	Molecular assay	Primer code	Sequence	References
*Phytophthora ramorum*	LAMP	Phy-r_F3	5′-ACGTTGTTGGTTGTGGAG-3′	This study
		Phy-r_B3	5′-CCAATTGAGATGCCAGCA-3′	
		Phy-r_FLP	5′-CGCATTGTTCAGCCGAAG-3′	
		Phy-r_BLP	5′-GAATCGACGGTGTTGTGC-3′	
		Phy-r_FIP	5′-AGTCATTACCGCCACAGCAGTGTTCGATTCGCGGTA-3′	
		Phy-r_BIP	5′-CGTAGCTGTGCAGGGCTTGAACCGCCACTCTACTTC-3′	
	qPCR	PramF	5′-GCAGGGCTTGGCTTTTGA-3′	Migliorini et al. (2018)
		PramR	5′-GCCGAACCGCCACTCTACT-3′	
		Pram_PR	5′-FAM-TCGACGGTGTTGTGCG-MGBNFQ-3′	
*Xylella fastidiosa*	LAMP	XF_F3	5′-TAGAGTCTTGGACTGAGCC-3′	This study
		XF_B3	5′-ATCGACCCAGTAATACTCGT-3′	
		XF_FLP	5′-AGGAGAACGTAATAACCACGG-3′	
		XF_BLP	5′-TCCTGGCATCAATGATCGTAAT-3′	
		XF_FIP	5′-CACCATTCAACATGGACTCGGTGCGATCTTCCGTTACCAG-3′	
		XF_BIP	5′-CTACGAGACTGGCAAGCGTTCGTACCACAGATCGCTTC-3′	
	qPCR	Xf_Fw	5′-CGGGTACCGAGTCCATGTTG-3′	This study
		Xf_Rev	5′-CAATCAAACGCTTGCCAGTCT-3′	
		Xf_Pr	5′-FAM-TGGTGCCCGTGGCTA-MGBNFQ-3′	
*Ceratocystis platani*	LAMP	CPL_F3	5′-CAGCGAAATGCGATAAGTAATG-3′	This study
		CPL_B3	5′-TTTATACTACACAGGGGAGTTG-3′	
		CPL_FIP	5′-AATGACGCTCGGACAGGCTCGAATCTTTGAACGCACA-3′	
		CPL_BIP	5′-TGTTCTTGGCGTTGGAGGTCGCAAGTATAACAGCCGATACA-3′	
		CPL_FLP	5′-TGCCTGGCAGAATACTGC-3′	
		CPL_BLP	5′-GTTCTCCCCTGAACAGGC-3′	
	qPCR	CpITS-F	5′-GCCTGTCCGAGCGTCATT-3′	Luchi et al. (2013)
		CpITS-R	5′-CCTCCAACGCCAAGAACAAA-3′	
		CpITS-Pr	5′-FAM-CACCACTCAAGGACTC-MGB-3′	
Cytochrome oxidase (COX) endogenous plant gene	LAMP	COX F3	5′-TATGGGAGCCGTTTTTGC-3′	Tomlinson et al. (2010a, b)
		COX B3	5′-AACTGCTAAGRGCATTCC-3′	
		COX FLP	5′-ATGTCCGACCAAAGATTTTACC-3′	
		COX BLP	5′-GTATGCCACGTCGCATTCC-3′	
		COX FIP	5′-ATGGATTTGRCCTAAAGTTTCAGGGCAGGATTTCACTATTGGGT-3′	
		COX BIP	5′-TGCATTTCTTAGGGCTTTCGGATCCRGCGTAAGCATCTG-3′	

### Real-time LAMP assay conditions

Real-time LAMP reactions were performed and optimised on the portable real-time fluorometer Genie^®^ II (OptiGene Limited, Horsham, UK). DNA samples were amplified for 30 min in Genie^®^ Strips (OptiGene Limited, Horsham, UK) with eight 0.2 mL isothermal reaction tubes with a locking cap providing a closed-tube system. Each isothermal amplification reaction was performed in duplicate, in a final volume of 25 μL. The reaction mixture contained 15 μL Isothermal Master Mix (ISO-001) (OptiGene Limited, Horsham, UK), 7 μL LAMP primer mixture (at final concentrations 0.2 μM of each F3 and B3, 0.4 μM of each FLP and BLP and 0.8 μM of each FIP and BIP) and 3 μL of template DNA. For each run two tubes including 3 μL dd-water were tested as No Template Control (NTC). LAMP amplification reactions were run at 65 °C for 30 min, followed by an annealing analysis from 98 to 80 °C with ramping at 0.05 °C per second that allow the generation of derivative melting curves (Abdulmawjood et al. 2014).

The main parameters used by Genie^®^ II system to assess the positivity of a sample are: amplification time (t_amp_) and amplicon annealing temperature (T_a_). The t_amp_ is the time (expressed in min) where the fluorescence second derivative of the signal reaches its peak above the baseline value, while the T_a_ is the temperature (expressed in °C) at which double-stranded DNA product dissociates into single strands.

### Specificity and sensitivity of real-time LAMP assays

For each target pathogen (*X. fastidiosa*, *C. platani* and *P. ramorum*) the specificity of the real-time LAMP assay was tested by using genomic DNA extracted from bacterial, fungal or oomycete strains (Table 1), at a final concentration of 10 ng μL^−1^. The limit of detection (LOD) of the LAMP assay was tested by using an 11-fold 1:5 serial dilution (ranging from 10 ng μL^−1^ to 0.001 pg μL^−1^) of each standard DNA template (*X. fastidiosa *- Co.Di.Ro strain; *C. platani *- isolate Cp24; *P. ramorum *- isolate Pram).

### Real-time LAMP assay in naturally infected plants

To check the suitability of extracted plant DNA for downstream analysis the cytochrome oxidase (COX) gene was used as endogenous plant gene according to Tomlinson et al. (2010a) (Table 2).

The effectiveness of the real-time LAMP assay was then tested on DNA extracted from naturally infected hosts (*Olea europaea*, *Rhamnus alaternus*, *Calicotome spinosa*, *Cistus incanus*, *S. junceum*, *Prunus dulcis*, *Platanus* × *acerifolia* and *Viburnum tinus*) to detect each respective target pathogen (*X. fastidiosa*, *C. platani* and *P. ramorum*). For each plant species, additional healthy plants DNA were also included as negative control.

### Real-time quantitative PCR assay

To validate the LAMP assay, for each target pathogen, DNA samples (from microbial and plant tissue) were also tested by real-time quantitative PCR (qPCR) based on TaqMan chemistry.

Primers and TaqMan^®^ MGB probe for the DNA quantification of *X. fastidiosa* with the StepOnePlus™ Real-Time PCR System (Applied Biosystems, Forster City, CA, USA) were designed using Primer Express™ 3.0 software (Applied Biosystems). The DNA sequence of the ribosome maturation factor (RimM) gene (CoDiRO strain) was obtained from the ‘National Center for Biotechnology Information’ (NCBI) (http://www.ncbi.nlm.nih.gov/entrez/query.fcgi) (accession number JUJW01000001). The TaqMan^®^ MGB probe was labelled with 6-carboxy-fluorescein (FAM) at the end, and a non-fluorescent quencher (NFQ) with minor groove binder (MGB) ligands, at the 3′ end. Primers and probe are reported in Table 2. The length of the amplification product was 60 bp. The identity of the amplicon sequence was determined by comparing with other fungal species with the Standard nucleotide–nucleotide BLAST (blast n) of the NCBI.

DNA samples were assayed in MicroAmp Fast 96-well Reaction Plates (0.1 mL) closed with optical adhesive and using the StepOnePlus™ Real-Time PCR System (Applied Biosystems).

The real-time PCR reaction was performed in a final volume of 25 µL. Each tube contained: 300 nM forward primer (Eurofins MWG Operon, Ebersberg, Germany); 300 nM reverse primer (Eurofins MWG Operon); 200 nM fluorogenic probe (Applied Biosystems); 12.5 µL TaqMan™ Universal Master Mix (Applied Biosystems); 5 µL DNA template.

Each DNA sample was assayed in three replicates. Four wells containing 5 µL sterile water each were used for a No-Template Control (NTC) without any nucleic acid. The PCR protocol was 50 °C (2 min); 95 °C (10 min); 40 cycles of 95 °C (30 s), 60 °C (1 min).

For each replicate the Ct value, defined as the point at which the Reporter fluorescent signal first became statistically significant against the background, was utilised to quantify the sample. Measurements of *X. fastidiosa* DNA in unknown samples were achieved by interpolation from a standard curve generated with a DNA standard (Co.Di.Ro. strain), which was amplified in the same PCR run.

Real time PCR protocols for *C. platani* and *P. ramorum* were those described in Luchi et al. (2013) and Migliorini et al. (2018), respectively.

### Statistical analysis

For each 1:5 serial dilution (ranging from 10 ng µL^−1^ to 0.128 pg µL^−1^) of each target pathogen, the correlation analysis was carried out between amplification time (t_amp_) for LAMP assay and threshold cycle (Ct) for qPCR.

## Results

### Specificity of real-time LAMP assay

For each target pathogen (*X. fastidiosa*, *C. platani* and *P. ramorum*) the nucleotide–nucleotide BLAST ^®^ search showed a complete homology (100%) between the LAMP amplicon sequences designed in the current study and the sequences of the same pathogen available in GenBank database (NCBI).

BLAST ^®^ search did not find sequence identity between the LAMP *X. fastidiosa* amplicon and the other species present in GenBank, while the *P. ramorum* LAMP amplicon showed 99% homology (due to only 2 bases of differences in the ITS region) with *P. lateralis* sequences. Similarly, the *C. platani* LAMP amplicon showed complete homology (100%) with *C. fimbriata* and 99% homology with *C. neglecta*, *C. ecuadoriana* and *C. manginecans*.

**Fig. 1:**
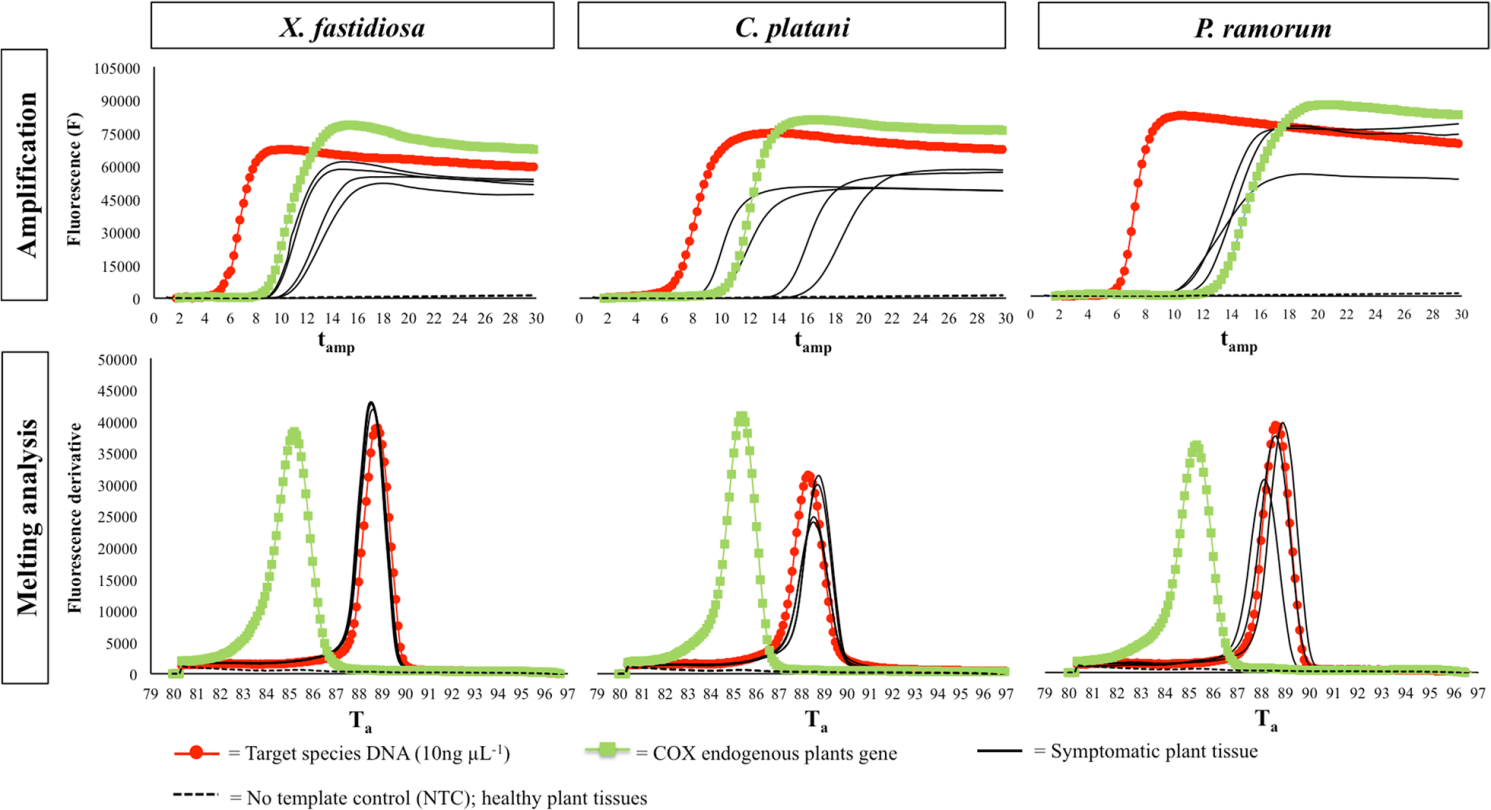
Selection of kinetics. Real time LAMP results reported as amplification and melting derivative plot for *Xylella fastidiosa*, *Ceratocystis platani* and *Phytophthora ramorum* including target species DNA (10 ng μL^−1^) in red, COX endogenous plant gene (green) and symptomatic plant tissues (black continuous line). No template control (NTC) and healthy plant tissue were also included (black dotted line). *T*_*a*_ annealing temperature, *t*_*amp*_ amplification time

LAMP assay was able to detect DNA of each target pathogen (*X. fastidiosa*, *C. platani* and *P. ramorum*) with positive results in the first time of the isothermal amplification (t_amp_ c.a. 7 min for *P. ramorum* and *X. fastidiosa*; c.a. 8 min for *C. platani*) (Fig. 1). All DNA samples of *X. fastidiosa* that include *X. fastidiosa* (Co.Di.Ro), *X. fastidiosa* subsp. *fastidiosa* (Xff) and *X. fastidiosa* subsp. *multiplex* (Xfm) were positively amplified by LAMP assay, and the melting curve showed a specific peak (T_a_ ranged between 88.78 and 88.98 °C) (Table 1). Bacterial DNA extracted from the other strains were not amplified by LAMP assay (Table 1). LAMP results were also confirmed by qPCR by using the designed primers (Xf_Fw and Xf_Rev) and probe (Xf_Pr) for *X. fastidiosa* (Tables 1, 2).

The real-time LAMP assay designed for *C. platani* was able to detect *C. fimbriata* strains belonging to different hosts and geographic origin (Table 1), whereas the qPCR assay gave negative results for these isolates. Similarly, the LAMP primers designed for *P. ramorum* were able to amplify *P. lateralis* DNA with melting temperatures very close to each other (Table 1). The other *Phytophthora* species included in this work either were not amplified or showed different amplification curves (with different t_amp_) or melting curves (with different T_a_) (Table 1). For each designed LAMP assay DNA from outgroup species and common host colonizer species were not amplified, as confirmed by qPCR (Table 1).

### Sensitivity of real-time LAMP assays

**Fig. 2:**
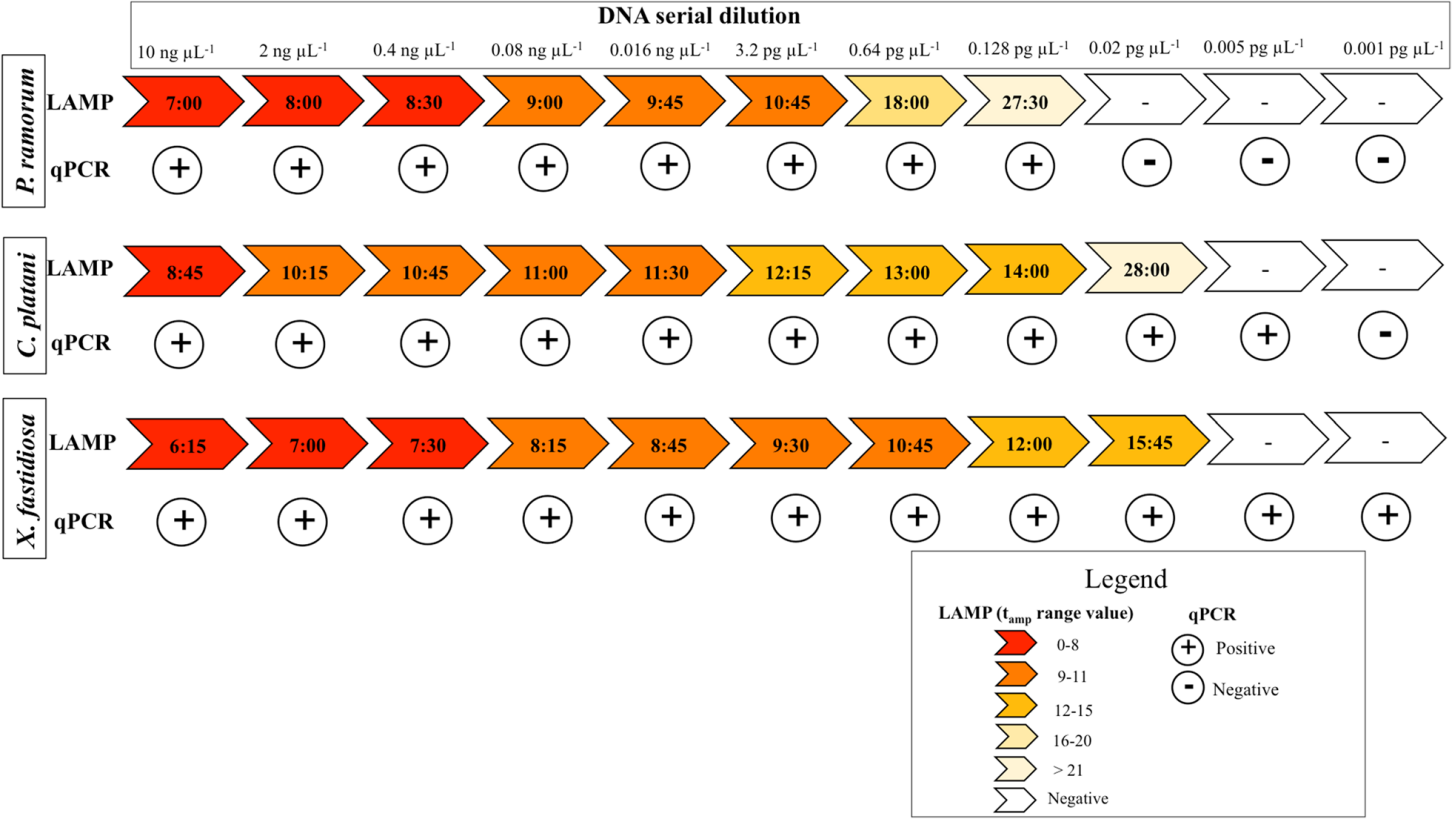
Sensitivity results obtained by testing both on LAMP and qPCR 11-fold 1:5 serial dilution (ranged from 10 ng μL^−1^ to 0.001 pg μL^−1^) of each standard DNA template (*X. fastidiosa*—Co.Di.Ro strain; *C. platani*—isolate Cp24; *P. ramorum*- isolate Pram). LAMP results are inserted in a scale from positive (red) to negative (white) based on amplification time (t_amp_; min:s). Real-time qPCR results are reported as positive (+) ore negative (−)

The values of limit of detection of LAMP assays (LOD_LAMP_) were always very low, ranging from 0.02 pg μL^−1^ for *X. fastidiosa* and *C. platani* and 0.128 pg μL^−1^ for *P. ramorum*, (Fig. 2; Table 3). *P. ramorum* qPCR assays had the same sensitivity as LAMP (LOD_qPCR_ = 0.128 pg μL^−1^). The qPCR assays for the other two pathogens were more sensitive than LAMP with lower detection limits (*X. fastidiosa*, LOD_qPCR_ = 0.001 pg μL^−1^; *C. platani*, LOD_qPCR_ = 0.005 pg μL^−1^) (Fig. 2).

**Table 3:** Comparison of different DNA extraction and LAMP protocols for *Xylella fastidiosa*, *Ceratocystis platani* and *Phytophthora ramorum* detection

Protocol	This study		Tomlinson et al. (2007)	Harper et al. (2010)	
DNA extraction					
Target pathogen	*Xylella fastidiosa*, *Ceratocystis platani*, *Phytophthora ramorum*		*Phytophthora ramorum*	*Xylella fastidiosa*	
Commercial kit	Plant Material Lysis Kit (OptiGene)	EZNA Plant DNA Kit (Omega Bio-tek)	QuickPick Plant DNA kit (Bio-Nobile)	Invimag Plant DNA Mini Kit (Invitek)	DNeasy Plant Minikit (Qiagen)
Use	Field	Laboratory	Field	Laboratory	Laboratory
Sample requirement	Fresh plant tissue (80–100 mg)	Fresh plant tissue (80–100 mg)	Fresh plant tissue (15–25 mg)	Lyophilized petiole (200 mg)	Fresh plant tissue (200 mg)
Advantages	Rapid and simple protocol with few reagents and steps; no laboratory instruments are required	Protocol kit with spin columns and buffer supplied	Processing up to 24 samples in parallel	Simplified sample processing	Grounding with beads; kit with spin column and buffer supplied
Disadvantage	Difficult for large number of samples	Required laboratories facilities for grinding and DNA extraction	Extremely basic equipment is needed	Required laboratories facilities for grinding and for DNA extraction	Required laboratories facilities for grinding and for DNA extraction
Time per sample	5 min	1 h	40–50 min	> 30 min	1 h
Isothermal DNA amplification					
Instrument	Genie II (OptiGene)		Smart Cycler (Cepheid)	ABI 9700 Thermocycler (Applied Biosystems)	
Use	Field		Laboratory	Laboratory	
Sensitivity (LOD)	*P. ramorum* (0.128 pg)		*P. ramorum* (10 pg)	–	
	*X. fastidiosa* (0.02 pg)		–	*X. fastidiosa* (1.4 pg)	
	*C. platani* (0.02 pg)		–	–	
Specificity	*P. ramorum* (high specific; *P. lateralis*)		*P. ramorum* (high specific; *P. lateralis*)	–	
	*X. fastidiosa* (very high specific)		*–*	*X. fastidiosa* (very high specific)	
	*C. platani* (high specific; *C. fimbriata*)		–	–	
Advantages	Rapid detection results; amplification and detection reaction is carried out in the same instrument (16 sample per run)		High number of samples to be processed	High number of samples to be processed	
Disadvantage	Strip tubes with amplification mix need to be prepared before in laboratory		Additional steps to visualize amplified products (electrophoresis gel, colorimetric detection, fluorescent dye)	Electrophoresis gel to visualize amplified products	
Time per sample	30 min		> 1 h	> 1 h	

*LOD* limit of detection

**Fig. 3:**
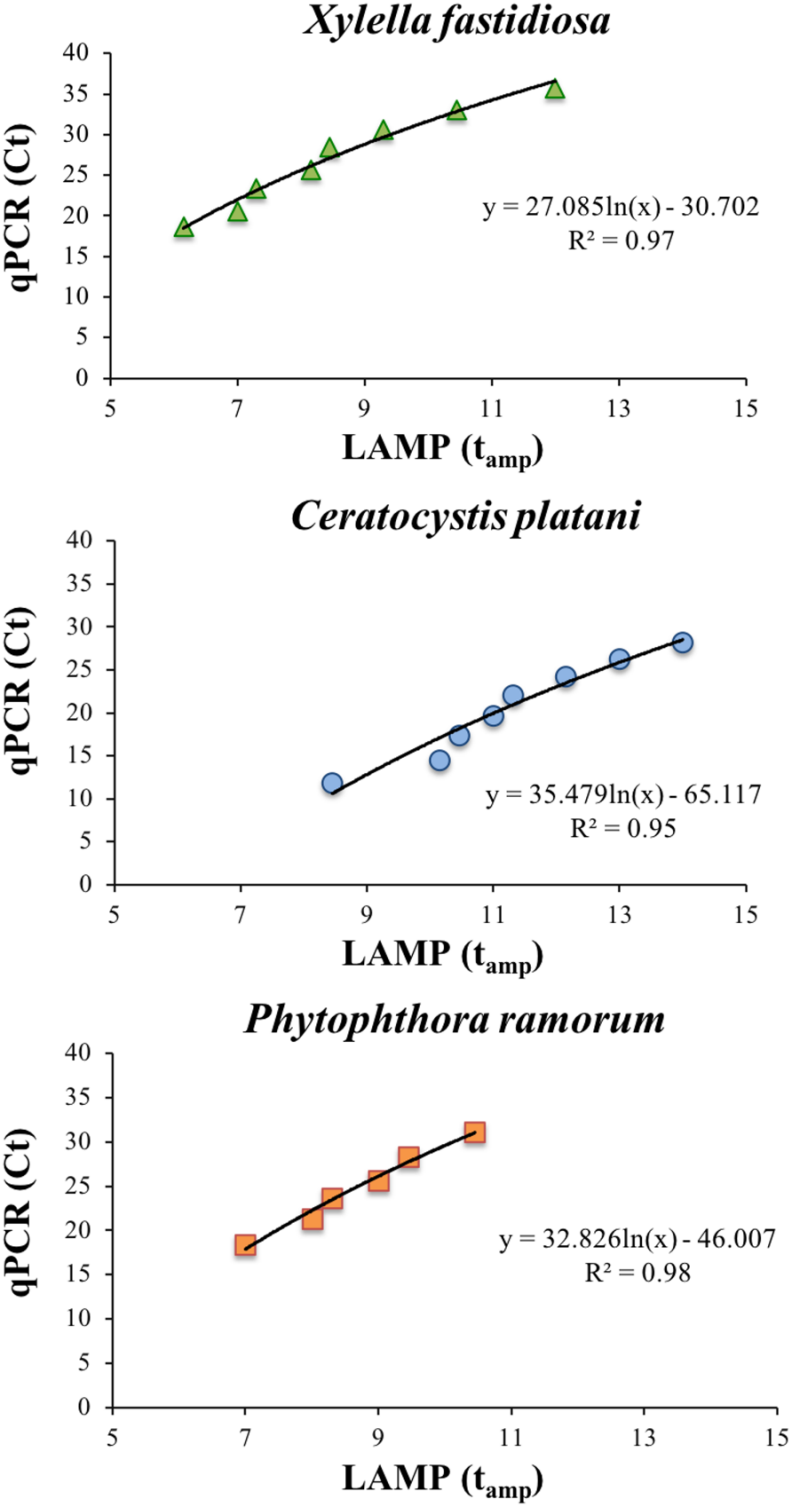
Statistical correlation between LAMP amplification time (t_amp_) and qPCR threshold cycle (C_t_) obtained by testing with both methods each 1:5 serial dilution (ranged from 10 ng μL^−1^ to 0.128 pg μL^−1^) of each standard DNA template (*X. fastidiosa*—Co.Di.Ro strain; *C. platani*—isolate Cp24; *P. ramorum*-isolate Pram)

We also observed a very strong correlation between the t_amp_ of the LAMP assay and Ct value of the qPCR in the same set of DNA samples (*X. fastidiosa*: R^2^ = 0.97; *C. platani* R^2^ = 0.95; *P. ramorum* R^2^ = 0.98) (Fig. 3).

### Real-time LAMP detection in plant samples

LAMP analyses carried out on plant host DNA were further validated by COX gene amplification, showing a specific melting peak at T_a_ = 85 °C for each analysed plant sample (both healthy and infected tissues) (Fig. 1). COX gene amplification was a reliable internal positive control, confirming host DNA extractions were successful by using both on-site DNA extraction kit (OptiGene) and laboratory commercial kit (Omega Bio-tek).

All symptomatic host plant samples (*Olea europaea*, *Rhamnus alaternus*, *Calicotome spinosa*, *Cistus incanus*, *S. junceum*, *Prunus dulcis*, *Platanus* × *acerifolia* and *Viburnum tinus*) were amplified successfully with the LAMP assay designed for each target pathogen (*X. fastidiosa*, *C. platani* and *P. ramorum*, respectively).

Symptomatic plant tissue showed similar T_a_ obtained from DNA of axenic cultures of each target pathogen (Table 1; Fig. 1), confirming the specificity of each LAMP assay to detect pathogens in infected plant tissues.

No amplification nor melting curve was obtained by applying the LAMP primers to healthy samples confirming the specificity of the LAMP optimized assay.

## Discussion

In this work LAMP assays for detecting *X. fastidiosa*, *P. ramorum* and *C. platani*, optimized for a portable instrument in real time were developed. LAMP-based assays optimized in this study allow a complete analysis (amplification and annealing) in only 30 min, starting to have positive amplification from ca. 7 min (Table 1). To our best knowledge no previous LAMP assay has been developed for *C. platani*. qPCR showed higher sensitivity with respect to LAMP in *X. fastidiosa* and *C. platani* detection, while for *P. ramorum* LOD was the same as that of LAMP.

The opportunity to have an accurate and rapid detection of the three quarantine pathogens considered in this study directly in the field by a portable instrument, represents a great advantage to preventing introductions and for applying control measures. Most of the LAMP-based assays recently developed for plant pathogens, including the one developed for *P. ramorum* by Tomlinson et al. (2007) and for *X. fastidiosa* by Harper et al. (2010), are based on laborious and time-consuming isothermal amplification reactions (Table 3). As an example, the LAMP protocol adopted by EPPO for *X. fastidiosa* detection and developed by Harper et al. (2010), requires ca. 60 min to amplify all the isolates tested by the author and to consistently amplify ca. 250 copies of template for reaction (corresponding to 1.4 pg μL^−1^ pathogen DNA) in host (*Vitis vinifera*) DNA. In comparison, the assay developed in the current study requires only ca. 15 min to amplify 0.02 pg μL^−1^ of *X. fastidiosa* DNA in dd-water. The use of a simple colour change method to assess the positive result of LAMP-tested samples (e.g. Hydroxynaphtal blue dye used in Harper et al. 2010), could be particularly suited for use in the field, but opening the tube to add the colorimetric dye makes the method extremely vulnerable to carryover contamination due to the very large amount of product generated by LAMP reaction (Tomlinson et al. 2007). Furthermore, some colorimetric dyes reagents can completely inhibit the LAMP reaction at the concentration needed to produce a colour change visible with the naked eye (Tomlinson et al. 2007) and even though they may be possible to observe in a laboratory environment, they are difficult to detect in the field due to the different light conditions at different times of the day (Lau and Botella 2017), leading to false negative results or to losses in detection sensitivity. In addition, the interpretation of positive results from colour changes in colorimetric dyes is very subjective, requiring experienced staff. On the contrary, the main parameters used to assess the positivity of a sample in a LAMP real-time assay, as the one developed in the present work, are amplification time (t_amp_) and annealing temperature (T_a_) resulting by fluorescence analysis results provided by the instrument.

The EPPO diagnostic protocol (PM 7/24) for *X. fastidiosa* describes a field LAMP assay based on the paper by Yaseen et al. (2015). In this paper authors optimized the Harper et al. (2010) assay for a portable instrument, but they do not report the sensitivity of the assay, strongly limiting its application due to the risk of false negatives.

LAMP assays developed in this study are specific and able to detect the target species, both from pure DNA and from DNA obtained from plant infected tissues. Some cross reactions have been observed in species genetically closely related to target species (for *C. platani*/*C. fimbriata* and *P. ramorum*/*P. lateralis*); however, their T_a_ is one-two degrees higher than that of the target organisms (89–90 °C vs. 88 °C), allowing a correct detection (Table 1).

A positive amplification sharing the same T_a_ of that of *P. ramorum* and *C. platani* (88 °C) was obtained only with *P. lateralis* and *C. fimbriata*, respectively. These species are almost morphologically indistinguishable and phylogenetically very close (De Beer et al. 2014; Kroon et al. 2012; Martin et al. 2014), but they were reported on very different hosts: *P. lateralis* attacks *Chamaecyparis* spp. and other *Cupressaceae* (Hansen et al. 2000; Robin et al. 2011), and *C. fimbriata* is the agent of sweet potato black rot (Okada et al. 2017).

The results of LAMP assays were also validated by those obtained from qPCR assays. The new TaqMan qPCR assay developed in this study for targeting *X. fastidiosa* is able to amplify all the *X. fastidiosa* tested subspecies with high efficiency excluding other tested bacteria species (Table 1). Furthermore, its sensitivity (0.001 pg μL^−1^) is much higher than that of the qPCR TaqMan assays developed by Harper et al. (2010) and by Francis et al. (2006) (both EPPO official diagnostic qPCR for *X. fastidiosa*) which has a detection limit of 0.05 pg μL^−1^, corresponding to 20 copies of template for reaction.

The use of rapid, specific and sensitive point-of-care methods like the LAMP assays developed in this study could enable phytosanitary services to make immediate management decisions, helping in containing environmental and economic losses. The application of such a portable diagnostic tool, requiring minimum equipment and a few, if any, specific scientific skills could be profitably used to check the health status of live plants or plant parts at the points of entry or in field, thus reducing time of analyses, thus allowing a prompt reaction. In conclusion, the results presented in this study show how an advance in technology can provide efficient tools to prevent the introduction or limit the spread of diseases that can have severe economic, ecological and sociological consequences.

## Abbreviations

*X. fastidiosa*: *Xylella fastidiosa*
*C. platani*: *Ceratocystis platani*
*P. ramorum*: *Phytophthora ramorum*
LAMP: loop mediated isothermal amplification
qPCR: real-time quantitative polymerase chain reaction
Ct: threshold cycle
t_amp_: amplification time
T_a_: amplicon annealing temperature
LOD: limit of detection
